# Prevalence of Asthma and Its Associated Risk Factors Among Children Aged 5–10 Years in a Rural Area of Coimbatore: A Community-Based Cross-Sectional Study

**DOI:** 10.7759/cureus.69329

**Published:** 2024-09-13

**Authors:** Surya Balakrishnan, Renuka Karunanidhi, Rathan Pandian, Sriram Ramamurthy, Sugunadevi Gurusamy, Thomas V Chacko

**Affiliations:** 1 Community Medicine, Panimalar Medical College Hospital & Research Institute, Chennai, IND; 2 Community Medicine, Karpagam Faculty of Medical Sciences and Research, Coimbatore, IND; 3 Community Medicine, Believers Church Medical College, Tiruvalla, IND

**Keywords:** asthma, children, isaac questionnaire, prevalence, risk factor, rural area

## Abstract

Introduction

Asthma is one of the serious health problems among children, accounting for a major proportion of lost school days due to absenteeism, decreased academic performances, and lesser social interactions. It can impair a child’s activities like playing sports and also their sleeping pattern with frequent school absenteeism. In South India, only a few school-based studies to determine the prevalence of asthma have been carried out, and even fewer studies are done in community settings in rural areas of Tamil Nadu. Hence, this community-based study was done to find out the prevalence of asthma among children aged 5-10 years in rural areas of Coimbatore, Tamil Nadu.

Aim & objective

To estimate the prevalence of asthma among children aged 5-10 years in rural areas of Coimbatore, Tamil Nadu.

Materials and methods

A community-based cross-sectional study was conducted in rural areas of Coimbatore, Tamil Nadu. A sample of 726 children from the randomly selected seven villages were studied. The sample size was calculated for a finite population. The standardized International Study of Asthma and Allergies in Childhood (ISAAC) questionnaire and calibrated instruments were used as data collection tools. Ethical approval was obtained. Data entry was done in Microsoft Excel and analyzed using the SPSS 19.0 version. The “t” test was used to compare the mean difference between the quantitative variables and the chi-square test was used to test the association between categorical variables. P-value <0.05 was considered as statistically significant.

Results

The overall prevalence of self-reported asthma was 5.1%. Boys had 1.7 times higher prevalence compared to girls. The prevalence of possible or suspected cases of asthma using the ISAAC screening questionnaire was 20.9%. In univariate analysis, the risk of having asthma among children is significantly higher among those with a positive family history of asthma.

Conclusion

The study found that the prevalence of self-reported asthma in rural settings is low (5.1%). However, our study also identifies a large number of children (20.9%) with symptoms suggestive of asthma using the ISAAC questionnaire that need the attention of a specialist for early diagnosis and care for reducing morbidity and episode severity.

## Introduction

Asthma is one of the major non-communicable diseases affecting all age groups of the population. Currently, about 262 million people are affected by asthma across the globe and this is likely to increase depending on different geographical and climatic conditions. According to the World Health Organization (WHO), asthma is caused due to inflammation and narrowing of airways in the respiratory tract. The disease is characterized by cough, breathlessness, chest tightness, and wheezing with varying frequency and intensity from one person to another [1].

Asthma is one of the most common diseases seen among children [1]. Globally, asthma poses a serious health problem, accounting for a major proportion of lost school days of absenteeism [2]. A child’s social interaction can be affected by asthma and also their academic performances [3]. Children’s activities like playing sports can be impaired by asthma. It also affects their sleep pattern and career success due to poor attendance in school [1,4].

Asthma is emerging as a major public health problem among children in low- and middle-income countries [1]. Despite advances in therapy, the prevalence, morbidity, and mortality of asthma are increasing [5]. Epidemiological studies showed that asthma-like symptoms are more prevalent in the community compared to physician-diagnosed asthma [6]. This underdiagnosis and underreporting of asthma have been seen particularly in children and young adults during the past two decades [7].

In spite of a large number of epidemiological studies, the etiology of asthma remains poorly understood. The occurrence of asthma among different age groups may be due to the interplay of both genetic and environmental exposures. Family history alone may not be a sufficient factor in the increased prevalence of asthma [8].

The prevalence of asthma and its risk factors shows a considerable regional variation. Understanding this geographical variation in the prevalence of asthma is important for gaining knowledge about epidemiological factors contributing to asthma and its etiopathogenesis and treatment modalities which would be helpful for treating physicians, epidemiologists, and also for community physicians [9].

Asthma cannot be cured completely but appropriate treatment can control the symptoms and may help people to lead an improved quality of life. Acute symptoms may be treated with short-term drugs while long-term medications like inhaled steroids are required to control the progression of severe asthma [1]. After an extensive literature search, it was found that most of the Indian studies were carried out in urban school-based settings in Northern parts of India. In South India, only a few school-based studies to determine the prevalence of asthma have been carried out, with a paucity of data regarding the prevalence of asthma obtained from community-based studies in rural areas of Tamil Nadu. Hence, this community-based study was done to find out the prevalence of asthma and its association with selected risk factors among children aged 5-10 years in rural areas.

## Materials and methods

Objectives

The objective of this study was to determine the prevalence of asthma among children in the age group 5-10 years in a rural area of Tamil Nadu and also to study the association of some selected risk factors with asthma.

Materials and method

Study Design and Setting

A community-based cross-sectional study was planned in the field practice area of the Rural Health Training Center of a private medical college which caters population distributed across 14 villages.

Sample Size

With an estimated prevalence of 10% from earlier studies [10,11] and 15% allowable error, sample size was calculated using the formula, n = (1.96)2pq/d2, where “n” represents sample size, “p” denotes prevalence from previous studies, “q” derived as (100 - p), “d” denotes allowable error. The calculated sample size is 1536. Since there were only 1220 children in the age group of 5-10 years in the field practice area, the sample size is calculated for finite population size [10,12] of 1220 children using the formula, n = n0/[1+(n0/N)], where “n” is new sample size, “n0” denotes sample size (1536), “N” represents the population size in the field practice area. Expecting a non-response rate of 5%, a final sample size of 715 is required. Out of 14 villages, seven villages were selected by lottery method till an adequate sample was obtained. As evident from the literature search, a high prevalence of asthma is seen among children in the age group 5-10 years.

Inclusion criteria

All the children both boys and girls in this age group from the selected seven villages were included in the study.

Study tool

After getting ethical approval (approval No: 13/390) from the institution human ethics committee (IHEC), a pilot study was carried out among 30 children in another rural area to identify difficulties in eliciting the data using the pre-validated instruments. Physical instruments used for measuring anthropometry were calibrated by the institution’s biometrics department. Anthropometric measurements of the children like height and weight were measured using standardized calibrated instruments as per WHO recommendations [13]. A semi-structured validated questionnaire was framed for collecting demographic details and a structured validated ISAAC (International Study of Asthma and Allergies in Childhood) questionnaire was used for eliciting asthma (ISAAC core questionnaire). Consent for participation in the study was obtained from a parent or guardian. The questionnaire was filled out by the parent or guardian who are literate. For others, the questionnaire was filled out by the principal investigator after eliciting the responses to the questions.

Data entry and analysis

Data was entered in Microsoft Excel and analyzed using the SPSS 19.0 version. The descriptive statistics (mean and standard deviation) were arrived at for the weight, height, and BMI of the children. The “t” test was used to compare the mean difference between the quantitative variables and the chi-square test was used to test the association between categorical variables. Univariate analysis was used to test the association with self-reported asthma. P-value <0.05 was considered as statistically significant.

## Results

Among 726 children studied, 357 (49.1%) were boys and 369 (51.9%) were girls. The mean (SD) height, mean (SD) weight, and mean (SD) BMI of the boys were 121.1 cm (7.16), 22.5 kg (5.77), and 15.11 kg/m^2^ (2.28), respectively. Similarly, the mean (SD) height, mean (SD) weight, and mean (SD) BMI of the girls were 119.9 cm (6.59), 21.1 kg (5.06), and 14.79 kg/m^2^ (2.19), respectively.

The primary objective of the study was to find the prevalence of asthma among children aged 5-10 years in the field practice area of the Rural Health Training Center (RHTC). Table 1 shows the age and gender-wise prevalence of self-reported asthma. Overall, the prevalence of asthma is 5.1%. Boys had 1.7 times higher prevalence compared to girls.

**Table 1:** Prevalence of self-reported asthma according to age and gender

Age (Completed Years)	Boys		Girls		Total no. of asthmatics (%)
	Total no. of subjects	No. of asthmatics (%)	Total no. of subjects	No. of asthmatics (%)	
5	87	5 (5.75)	91	5 (5.5)	10 (5.62)
6	57	5 (8.77)	72	2 (2.78)	7 (5.43)
7	64	7 (10.94)	63	2 (3.18)	9 (7.09)
8	51	1 (1.96)	62	2 (3.23)	3 (2.66)
9	75	5 (6.67)	58	3 (5.17)	8 (6.02)
10	23	0	23	0	0
Total	357	23 (6.44)	369	14 (3.79)	37 (5.1)

Table 2 shows the overall prevalence of suspected or possible cases of asthma was 20.9% and this is slightly higher among boys (21.8%) compared to girls (20.1%).

**Table 2:** Prevalence rate of suspected or possible cases of asthma according to gender using ISAAC questionnaire for screening ISAAC: International Study of Asthma and Allergies in Childhood

		Asthma		Total Number (%)
		Yes	No	
		Number (%)	Number (%)	
Gender	Boys	78 (21.8)	279 (78.2)	357 (49.2)
	Girls	74 (20.1)	295 (79.9)	369 (50.8)
Total		152(20.9)	574 (79.1)	726 (100)

Table 3 shows the distribution of suggestive symptoms of asthma among children in the study group. Out of 726 participants, 87 (12%) had a history of wheezing. In both the age groups (5-7 years and 8-10 years), prevalence was higher among boys but not statistically significant.

**Table 3:** Distribution of suggestive symptoms of asthma in the study group by age and gender

Symptoms	5-7 years (n=434)			8-10 years (n=292)			Total (n=726)
	Boys (n=208)	Girls (n=226)	p-value	Boys (n=149)	Girls (n=143)	P-value	N (%)
	N (%)	N (%)		N (%)	N (%)		
History of wheezing	34 (16.3)	29 (12.8)	0.299	14 (9.4)	10 (7)	0.455	87 (12)
History of wheezing in last 12 months	25 (12)	18 (8)	0.158	11 (7.4)	7 (4.9)	0.377	61 (8.4)
Frequency of wheezing (1 or more times) in last 12 months	22 (10.6)	13 (5.8)	0.065	11 (7.4)	6 (4.2)	0.377	52 (7.2)
Frequency of sleep disturbance (1 or more times) in last 12 months	15 (7.2)	12 (5.3)	0.210	7 (4.7)	5 (3.5)	0.334	39 (5.4)
Speech disorder due to wheezing in last 12 months	13 (6.3)	11 (4.9)	0.276	6 (4)	4 (2.8)	0.164	34 (4.7)
History of asthma	17 (8.2)	9 (4)	0.066	6 (4)	5 (3.5)	0.812	37 (5.1)
History of wheezing due to exercise in the last 12 months	21 (10.1)	17 (7.5)	0.253	12 (8.1)	9 (6.3)	0.729	59 (8.1)
History of nocturnal dry cough in last 12 months	40 (19.2)	35 (15.5)	0.303	19 (12.8)	24 (16.8)	0.331	118 (16.3)

History of wheezing during the last 12 months was positive in 61 (8.4%) children and higher among boys in both age groups when compared to girls. However, the difference was not statistically significant. Fifty-two (7.2%) children experienced at least one time wheezing in the last 12 months and boys had a higher prevalence when compared with girls. However, the difference was not statistically significant. Regarding sleep disturbance in the last 12 months, 39 (5.4%) children had suffered at least one episode of sleep disturbance. This prevalence was higher among boys in both age groups. However, the difference was not statistically significant. Speech limitation in the past 12 months was reported in 34 (4.7%) children. This was higher among boys in both age groups when compared to girls. However, the difference was not statistically significant.

A history of asthma was found in 37 (5.1%) children. Among boys, prevalence was 8.2% and 4% in the two age groups. This prevalence was higher than girls 4% and 3.2% in the two age groups respectively. However, the difference was not statistically significant.

Overall, 59 (8.1%) children reported a history of wheezing followed by physical exercise in the last 12 months. It was 10.1% among boys in the age in the age group 5-7 years to 7.5% in girls. Similarly in the other group, this is higher among boys (8.1%) than the girls (6.3%). However, the difference observed between both sexes in each group was not statistically significant.

Among 726 children, 118 (16.3%) children had dry cough during past 1 year. This difference was not statistically significant among boys and girls in both groups. The difference observed among boys and girls was 19.2% and 15.5% in 5-7 years group. Similar value for other group was 12.8% and 16.8% respectively.

Table 4 shows the mean BMI of children with asthma is slightly lower than children without asthma and this difference is not statistically significant.

**Table 4:** Association of BMI with asthma

	Presence of asthma	No. of children	Mean	Standard deviation	t-value	p-value
BMI	Yes	37	14.61	2.14	0.516	0.606
	No	689	14.80	2.19		

Table 5 shows that the risk of having asthma among children is significantly higher among those with a positive family history of asthma and not significant with the gender of the child.

**Table 5:** Univariate analysis to identify factors associated with self-reported cases of asthma *Statistically significant p-value <0.05

Factors	Category	Total	Asthma		Unadjusted odds ratio	95% confidence interval	p-value
			Number	%			
Gender	Boys	357	23	6.4	1	1	
	Girls	369	14	3.8	0.573	0.29-1.132	0.105
Family history of asthma	No	560	11	2	1	1	
	Yes	166	26	15.7	9.26	4.47-19.21	<0.001^*^

## Discussion

The study was undertaken to find out the prevalence of asthma among children aged 5-10 years in the field practice area of an RHTC attached to a private medical college and to identify the possible association of selected risk factors like family history of asthma, gender of the child and BMI with asthma.

For this purpose, a total of 726 children in the age group of 5-10 years were studied for the prevalence of asthma from the seven selected villages. A validated standardized ISAAC core asthma questionnaire was used as the study tool to find out the proportion of children with symptoms suggestive of asthma. Anthropometric measurements of the children like height and weight were measured using standardized calibrated instruments as per WHO recommendations [13]. The questionnaire also included demographic details of children which were obtained from parents during the time of interview. For the purpose of finding the prevalence of asthma child was labeled to have asthma, if the answer by the parent/guardian was “yes” to the question “Did the child suffer from asthma”. The child was labeled as a suspected or possible case of asthma if the answer by the parent/guardian is “yes” to any one of the eight ISAAC questions.

Estimates of the prevalence of asthma across various parts of India vary based on geographic variations, seasonal variations, etc. In our study, the overall prevalence of asthma was 5.1% which is similar to Chakravarthy et al [14]. Few studies like Asher et al. [15], Awasthi et al. [16], and Rashmi et al. [17] quoted the prevalence of asthma as less than 5%. While study done by Kamath et al. [18] and Kulkarni et al. [19] reported 6.3% prevalence.

In our study, there was no statistically significant difference in the prevalence of asthma between boys and girls. These findings are at variance with various other studies like Jain et al. [10] and Cheraghi et al. [20] have reported a higher prevalence of asthma among boys. The mean BMI of children with asthma was 14.61 which was slightly lower than children without asthma in whom it was 14.80 and this difference was not statistically significant. A study done by Corbo et al. [21] has reported a positive association between elevated BMI and asthma.

Family history of asthma in any one of the parents or grandparents played a major role in the development of asthma in children. This risk increased with both parents and grandparents having asthma. Family history of asthma was present in 166 children of our study population, among those children, 26 (15.7%) were found to have asthma. This difference was statistically significant and is consistent with studies done by Jain et al. [10], Cheraghi et al. [20], and Vyankatesh et al. [22].

Strengths and limitations

This study was a rural community-based study since many similar studies were done in urban school settings. The study used a standard validated questionnaire and equipment. The age group in our study was between 5 and 10 years, so the results may not be generalizable to children beyond this age group. The sample size for the study was determined to arrive at the prevalence of asthma. The same is not applicable to studying the association of asthma with the selected risk factors.

## Conclusions

The study revealed that the self-reported prevalence of asthma was low. While using the ISAAC core questionnaire, the prevalence of suspected or possible cases of asthma was high when compared to the self-reported prevalence of asthma. Hence, the health workers can use the ISAAC core asthma questionnaire to identify those children who need the attention of a physician and initiate treatment and counseling at the earliest.

## Appendices

Questionnaire for parents/guardians

Demographic Details

Name                                                                    ID No 

Age                                                                       Gender              

DOB                                                                                            

 Weight (kg)                                                  Height (cm)

Informant:          Mother/Father/Others (specify) _______________

Total no. of family members                Total monthly family income                                                  

Father’s Age (Yrs)                                            Mother’s Age (Yrs)

 Father’s educational status                                 Mother’s educational status

 Illiterate                                                                 Illiterate                                              

Primary                                                                 Primary

Secondary                                                            Secondary

Higher secondary                                                Higher secondary

Graduate                                                               Graduate

Does child’s parent/grandparents suffer from asthma? Yes / No

ISAAC questionnaire

1.     Did your child suffer from difficulty in breathing with wheezing or whistling sound?

                   Yes                                      No

        If No, skip to question no. 6.

2.     In the past 12 months did your child had whistling or wheezing with difficulty in breathing?

                    Yes                                      No

        If No, skip to question no. 6.

3.     During past 12 months, how many times did your child had wheezing problem?

                 Never                                  1 - 3 times 

                 4 - 12 times                         > 12 times

4.     During last 12 months, how many times did your child wake up due to wheezing during sleep?

        Never                   <1 night in a week                  >1 night in a week

5.     During last 12 months, was your child unable to speak more than 1 or 2 words due to wheezing?

                  Yes                                      No

6.     Did the child suffer from asthma?

                  Yes                                      No

7.     During last 12 months, during exercise did your child have wheezing?

                  Yes                                      No

8.     During last 12 months, did your child had dry cough during night other than upper respiratory tract infection?

                  Yes                                      No
